# Methodological Considerations for Movement Education Interventions in Natural Environments for Primary School Children: A Scoping Review

**DOI:** 10.3390/ijerph19031505

**Published:** 2022-01-28

**Authors:** Luca Petrigna, Ewan Thomas, Antonino Scardina, Federica Rizzo, Jessica Brusa, Giovanni Camarazza, Claudia Galassi, Antonio Palma, Marianna Bellafiore

**Affiliations:** 1 Sport and Exercise Sciences Research Unit, Department of Psychology, Educational Science and Human Movement, University of Palermo, Via Pascoli 6, 90144 Palermo, Italy; ewan.thomas@unipa.it (E.T.); ninoscardina93@msn.com (A.S.); r.federica1991@gmail.com (F.R.); brusajessica@gmail.com (J.B.); giovanni.caramazza@unipa.it (G.C.); claudia.galassi@unipa.it (C.G.); antonio.palma@unipa.it (A.P.); marianna.bellafiore@unipa.it (M.B.); 2 Regional School Office of Sicily (USR Sicilia), Via San Lorenzo Colli n° 312/g, 90146 Palermo, Italy

**Keywords:** outdoor movement education, natural environment, academic achievement, primary education

## Abstract

Background: Education is the ideal setting for carrying out projects to improve primary students’ capacities. In recent years, interventions in natural environments have been more frequently proposed, but there is still a lack of standardization, making deeper study of the topic necessary. This review aims to report on what previous scientific research has been carried out, and eventually, to propose standard operating procedures for future interventions. Methods: This is a scoping review that adopted the PRISMA guidelines. Primary school children have been included, and the interventions had to be proposed adopting nature as the primary element of the learning process. Results: A total of 19 studies have been included, and a wide range of methodological differences has been detected regarding the included intervention protocols. Conclusions: Learning in nature is a feasible intervention that, despite the high heterogeneity of interventions, demonstrates positive outcomes in the learning sphere of children.

## 1. Introduction

School is an ideal environment for carrying out long-term projects due to the huge amount of time children spend in this setting [1,2]. Fortunately, education is changing, by adopting active learning and other kinds of outdoor interventions that can improve the learning sphere [3]; however, further improvements are still required [4]. Some studies have highlighted how a green schoolyard may improve children’s cognitive and social well-being [5]. A conventional school is ideal for playing vigorous, competitive, rule-bound games. This, however, has been found to limit girls’ play, while active play across different interests and abilities should be promoted. A green design can help this process [6]. Conventional schools, with fences and barren flat surfaces, limit the opportunities to be physically active. Conversely, green spaces promote physical and social health [7], making nature interesting for interventions regarding physical and mental skills.

Interventions in nature are a valid approach with numerous, wide-ranging benefits for young children in regard to learning [8]. Spontaneous outdoor, deliberate play remains in children’s minds and effects inhibitory efficiency [9]. Nature, consequently, could become an arena for learning some academic subjects through the experience of movement [10]. Interventions in nature create challenges and opportunities of various types. Sensory stimulation combined with increasing the playing area allows for contact with different materials, thus promoting physical and social connections and open movement solutions [11]. However, despite findings that free play may increase engagement, results have indicated that it is insufficient as an intervention. Instead, children should be invited into physical activities [11], which makes structured interventions necessary.

The standardization of these interventions should, therefore, be considered to allow for comparisons between studies. The creation of normative data and the prevention of misconduct, mistreatment, or potential legal or ethical issues, should be widely implemented, especially among children. Therefore, the adoption of standard operating procedures is strongly suggested [12,13]. Standard operating procedures are documents that provide details of a process, allow for the correct repetition of all steps [14,15], and are widely adopted across many disciplines, such as in nuclear power plants, hospital emergency care, the diagnosis and treatment of pathologies, and aviation, where standardization is fundamental [13,14].

Considering the positive aspects of learning in nature, the focus of this review is to investigate and synthesize the protocols of structured interventions occurring in natural settings among primary school children and provide an overview of the effects on the learning sphere. Secondly, we propose a standard operating procedure.

## 2. Material and Methods

This scoping review of literature partially adopted the Preferred Reporting Items for Systematic Reviews and Meta-Analyses extension for Scoping Reviews (PRISMA-ScR) checklist and explanation [16].

### 2.1. Eligibility Criteria

The eligibility criteria for Population, Intervention, Comparison, Outcomes, and Study design (PICOS) were considered.

The included studies had to include the population of children between the ages of 3 and 12 years who were attending primary/elementary school. Studies that only included students with physical and mental disabilities were excluded due to the specific focus of the interventions.

The interventions had to include outdoor learning in a natural setting. Outdoor learning, in this review, is considered to be a structured lesson, performed on school grounds (i.e., not regular sports grounds), during which the teachers incorporated specific curricular subjects. A natural environment was considered to be a forest or a woodland area, as well as a park or a garden. The intervention had to be carried out during school hours. Interventions taking place in local neighborhoods or museums, day excursions, and camp expeditions were excluded. Curricular physical education, physical activity breaks, recess, and after-school interventions were also excluded.

Considering the objective of this review, which was to focus attention on the protocols used, no limitations were adopted for the “comparison” section. The considered outcomes were the improvement of physical activity, academic achievements, and social behaviors.

The study designs included were cross-sectional, interventions, observational, longitudinal, and correlational (including randomized and non-randomized controlled and quasi-randomized studies). Case studies were excluded. Only peer-reviewed English articles were included due to the language knowledge of the authors. Reviews and meta-analyses, abstracts, citations, opinion articles, books, book reviews, letters, editorials, statements, and commentaries were excluded.

### 2.2. Data Collection

The systematic search was performed through the electronic databases of PubMed, Web of Science, Scopus, and Education Resources Information Center (ERIC), and included studies published up until 1 September 2021, which corresponds with the date of the article search.

The keywords were divided into three groups and were combined through Boolean operators. These groupings and the full search strategy are presented in the appendix for clarity.

### 2.3. Study Record

The manuscripts detected in the electronic databases were included in EndNote software (EndNote version X8; Thompson Reuters, New York, NY, USA). In the first step, duplicates were searched for. After this, two independent investigators screened the manuscripts against the eligibility criteria based on the title, abstract, and full-length article. If the two investigators were in disagreement in selecting a manuscript, the coordinator of the study was involved in providing the tie-breaking decision. None of the investigators were blinded to the authors or associated institutions of the manuscripts during the selection process.

Information related to the study (design and location), sample characteristics (sample size, age, and gender), intervention protocols (type, duration, and frequency), outcomes, and main conclusions, were collected. The data were discussed narratively and represented through tables.

## 3. Results

**Figure 1:**
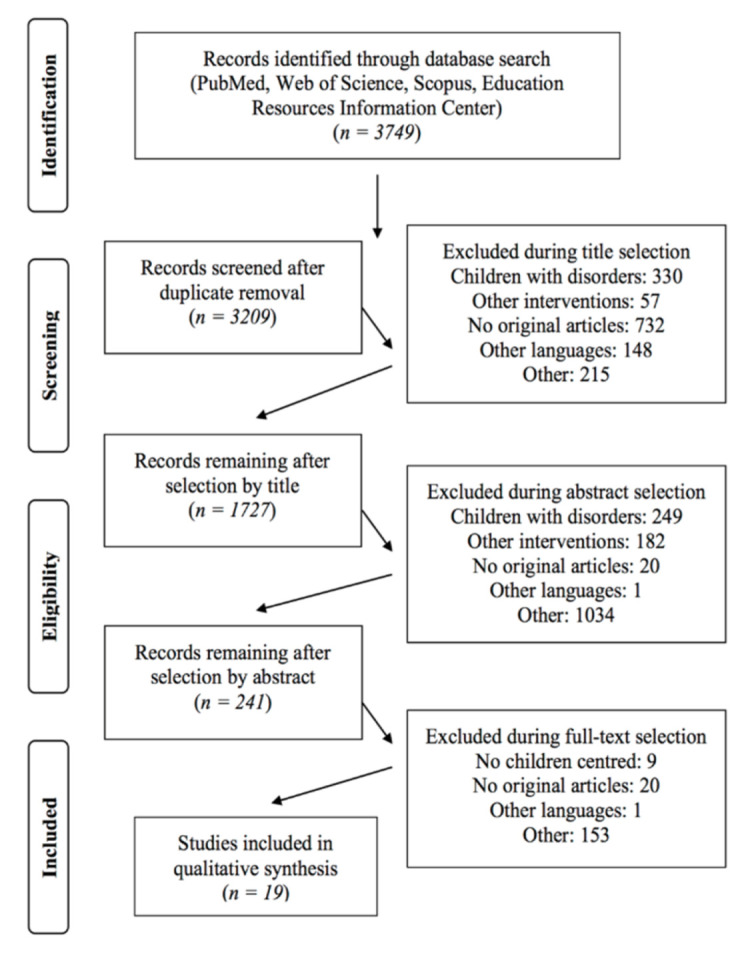
Flow diagram representing the selection process for manuscripts.

A total of 3749 papers were retrieved after the screening process (PubMed: 2823; Web of Science: 358; Scopus: 283; ERIC: 285); of these, 540 were duplicates. After the screening process based on the title, abstract, and full-text, a final number of 19 papers were included in the review. The PRISMA flow diagram presented in Figure 1 represents the selection process for the manuscripts. Within the figure, the manuscripts excluded against the eligibility criteria deemed as “other” were all those papers that included a population of different ages (differing from the inclusion criteria) or with particular diseases (such as cancer, stroke, amputees, or physical or mental disabilities), or whose interventions were not proposed in a natural environment, taking place during school hours, or did not include movement as primary component (as determined during full-text selection; see Figure 1).

### 3.1. Study Characteristics

The included studies were published between 2006 and September 2021. These studies were carried out in Denmark (*n* = 7), the United Kingdom (*n* = 4), Turkey (*n* = 3), the United States of America (*n* = 2), India, Germany, and Finland (*n* = 1). A quasi-experimental design was the most commonly adopted methodology (*n* = 6), but authors also adopted prospective studies (*n* = 2), mixed-methods intervention studies (*n* = 2), matched-groups, cross-sectional study, pilot study, qualitative study, triangular design, and observation study (*n* = 1).

A total of 4587 children were included across the selected studies. Of these children, 1553 of them were female (34%), 1344 were male (29%), and for 1690 children, gender was not specified (37%). The mean age (and standard deviation) was 9 (2.6). For more details, see Table 1.

### 3.2. Intervention Characteristics

The most frequently adopted project was the TEACHOUT/“Education outside the classroom” model (EOtC, *n* = 7); this was followed by the Forest School project (*n* = 4), and Wilderness Schooling was only used in one study (*n* = 1).

The mean length of the interventions was 162 (SD = 75) days, and they ranged from 42 to 180 days. Most of the studies proposed interventions that were performed twice a week (*n* = 5), but these ranged from one to six times a week. The mean amount of time per week was 9 (SD = 8) hours with a range of 1–25 h.

The interventions aimed to evaluate physical activity levels through accelerometers (*n* = 5), the Physical Activity Questionnaire (PAQ-C), and the Child Diary Report of PA Periods and School Day Activities.

Learning performance was evaluated through reading performance and reading speed, a comprehension test, drawings abilities, academic attainment scores, and written and scientific assessments. The Draw an Environment Test Rubric (DAET-R), the Academic Self-Regulation Questionnaire (SRQ-A), the Minnesota Comprehensive Assessments in Math and Writing, the Affective Self-Report, and the Skills Self-Report were also adopted.

The social sphere was evaluated through social relations, perceived exploration, and collaboration using the Danish Occupational Social Class (DOSC) measurement scale. Authors also adopted the Strengths and Difficulties Questionnaire (SDQ) and the stress level test for the evaluation of stress. Heart rate variability and the d2 test were also employed. In one study, the incidence of illnesses and injuries were evaluated.

All studies adopting the EOtC project taught academic skills and concepts in an illustrative way, such as calculating a tree’s volume using math (in forests, nature schools, or school grounds), teaching language skills through poem writing, and teaching history or religion while visiting places of historic significance (such as museums of farms) [36].

All projects that adopted the Forest Schools project aimed to connect children with nature and involved spending part of the classroom time in natural spaces during the school day. The educational style of Forest Schools is informal (structured at the beginning and becoming unstructured with a play-based program), and the principles are the development of self-confidence and self-esteem; behavioral, social, and emotional wellbeing; physical health; and awareness of and respect for the natural environment. The Forest School is usually a long-term intervention that takes place in a woodland or wooded context and aims to achieve the holistic development of the child. This methodology is performed by qualified practitioners and is a child-centered process [37].

The intervention proposed by Romar and colleagues [31] was a mix of udeskole and Forest School learning concepts, and it consisted of free and teacher-organized play, cooperative learning tasks, orienteering, a sloyd (woodwork) task to carve pencils from materials found in the forest, reading the workbook, and studying animals and plants in the forest.

Other studies [17,29] proposed different activities during the forest visit, such as free play, art, music, and story-telling activities, and semi-structured lessons for science and mathematics. Free choices and walks in the forest were also proposed [38]. The intervention carried out by Khan and colleagues [25] acted on the school ground and was a combination of seven behavioral settings (natural learning, water learning, loose materials, amphitheaters, and play areas, as well as gardens and huts) which they used to teach science and mathematics. English reading and writing, science, and math were taught outdoors in a natural setting [23,30]. Similarly, activities to improve cognitive, motor, linguistic, social, and emotional development were outside [35]. In one last study, children had to explore the natural environment, photograph and identify animal species, and take notes about the appearance and location of the organisms found [33].

### 3.3. Intervention Outcomes

School days spent in nature increased daily physical activity levels [32,34] and motor skills [35] and decreased sedentary behaviors [21]. Children’s learning was improved [30,33]; in particular, cognitive and affective outcomes [23] were improved, with positive associations with linguistics [35] and reading [28]. Academic attainment was also improved [25]. From a social perspective, education in nature promoted social well-being [19] and was also positively associated with gaining new peers [20].

## 4. Discussion

This scoping review highlights that different kinds of interventions in nature were proposed and that these function in different ways and with different modalities (see Table 1). For this reason, a standard operating procedure has been proposed (Table 2). Furthermore, the results suggest that learning interventions in nature have positive effects on the learning spheres of children.

Some projects carried out structured lessons that integrated different curricular subjects into outdoor interventions. Others directly addressed curricular subjects in a forest and made forest exploration a way for children to learn, and still others used school grounds as academic spaces. Examples of these interventions are the udeskole concept and the Forest School. A form of outdoor teaching is the udeskole (which, in English, could be translated as “out-of-school-teaching”) concept, which has been widely adopted in Denmark, Norway, and Sweden [36]. Udeskole uses local and outdoor environments to contextualize cross-disciplinary learning in concrete experiences, providing authenticity to the learning process and promoting curiosity [37]. This is an intervention that has presented a limited formal research, even if it has shown positive effects on the health, social functioning, and well-being of people [36]. The Forest School concept, which was first adopted in the United Kingdom in 1993, is increasing in popularity, thus outlining the interest in these programs [39]. The Forest School aims to foster relational and meaning-making opportunities with nature connectedness and promotes health, wellbeing, and pro-environmental outcomes [40]. Forest Schools, such as outdoor learning programs, seem to facilitate children’s growth acting through the promotion of intrinsic motivation and positive functional outcomes, as suggested by self-determination theory [41]. These allow for constructive learning, meaning that children learn and understand through their actions, and this remains throughout the rest of their lives [39].

Despite the differences in the protocols and project structures, positive outcomes have been provided by the authors regarding intrinsic school motivation [18], with positive academic [24,26,27,31], cognitive, and affective outcomes [23]. A decrease in sedentary behaviors and an increase in physical activity levels [21,32,34] have been also been highlighted. Social well-being [19] and gains in new peers [20] were noted in one study, which detected that the performance of the classroom in a forest also declined cortisol levels [38]. Finally, a natural school ground design increased children’s academic attainment [25]. All these positive outcomes could be explained by children being more intrinsically motivated to learn during the intervention in nature [40]. This has important similarities with the principles of scouting, which aims to promote the learning of skills by adopting nature as a school [42].

An additional aspect is that nature interventions among children can be performed everywhere; indeed, there are studies from Scandinavian countries and Turkey, the United Kingdom, and the United States of America, as well as India and Germany. Other studies were also performed in Australia and New Zealand, where the harsher climate and dangers due to venomous and/or poisonous flora and fauna could represent limiting factors [43].

One limitation regarding the adoption of Forest Schools is the relatively high cost, which could represent an obstacle in some contests; the main limitation of udeskole is that it sometimes requires movement to specific locations and it requires more than one teacher to control the students while in the environment itself [37]. Furthermore, teachers underlined that these interventions require a specific preparation and a considerable amount of time to plan [10].

In an ideal intervention in nature, the following are required: (a) children should feel safe to enact self-directed behaviors; (b) children should be allowed to rest and hide within the place as they wish; (c) ownership of spaces should be promoted; (d) children should be enabled to manage their own risk [44]. Furthermore, as Bentsen and colleagues suggest, a collaboration between researchers, local governments, and educational and landscape planners is necessary to improve these programs [36].

This review presents different limitations. First, the literature included has been published only in peer-reviewed journals, thus reducing the number of studies included and limiting our overview to only some of the existing papers. However, including only peer-reviewed articles increases the quality of this scoping review. A second limitation is the impossibility of performing the review as a systematic review or meta-analysis, due to the range of different outcomes and study designs of the included studies. Future studies should delve deeper to evaluate the effects of interventions in nature on academic subjects through the use of standardized and specific tests. Furthermore, studies based on learning interventions in nature should also be performed in other parts of the world to determine the feasibility of such interventions in different natural environments.

## 5. Conclusions

Learning in nature is a feasible intervention that has positive outcomes in the learning sphere of children. Unfortunately, the literature on the topic is limited; therefore, a standard operating procedure has been proposed. This topic requires further attention, and future studies should concentrate on proposing structured interventions among primary school children in nature that also include curricular subjects.

## Figures and Tables

**Table 1:** Characteristics of the included studies.

Author, Year	Nationality	Projects	Study Design	Number (F) “M”	Age (Years)	Length (Days)	h/Week	Times/Week	Evaluation Methods	Conclusions
Ahi, 2021 [17]	Turkey	Forest Kindergarten	Triangular design	35	4–6				DAET-R	Children in forest schools often meet with nature
Bølling, 2018 [18]	Denmark	Teachout/EOtC	QE	618	8–14	365	2–7	2	Academic Self-Regulation Questionnaire	EOtC is associated with increased intrinsic school motivation
Bølling, 2019 [19]	Denmark	Teachout/EOtC	QE	733	10–12	365	2–7		SDQ; DOSC measurement	EOtC promotes social well-being
Bølling, 2019 [20]	Denmark	EOtC	QE	448 (250) “198”	8–14	180	5	2	Social relations	EOtC was positively associated with a gain in new peers
Bølling, 2021 [21]	Denmark	Teachout/EOtC	QE	617 (341) “276”	10.9	180	5	1-2	Acc; Child Diary Report of PA Periods and School Day Activities	School days with green EOtC must be considered promising to counteract sedentary behaviors
Dettweiler, 2017 [22]	Germany		Prospective longitudinal	48 (18) “30”	11.2	365	1		Acc; stress levels	A steady decline of cortisol during the school day in the forest
Ernst, 2006 [23]	USA		Observation	50	11	180	12	6	Minnesota Comprehensive Assessments; Affective and Skills Self-Report	Results suggest positive cognitive and affective outcomes
Frenkel, 2019 [24]	USA		Prospective cohort study	141 (58) “83”	2–5	98	25	5	Incidence of illness/injury	Nature preschools are a healthy and safe child-care model
Khan, 2020 [25]	India		Mixed-methods	123 (61) “62”	8–11	150	8	6	Academic attainment; exploration and collaboration	School grounds contribute to improvements in academic attainment
Marchant, 2019 [26]	UK	Forest School	Qualitative	75 (47) “28”	5–6				Qualitative assessment	OL as a curriculum-based program is supported
Mygind, 2018 [27]	Denmark	EOtC	Pilot	47	10–12	90			Heart rate variability; d2 test	Natural environments may give rise to stress-buffering influences
Otte, 2019 [28]	Denmark	Teachout/EOtC	QE	529 (292) “237”	11	180		2	Reading performance/comprehension	EOtC is positively associated with reading
Pamuk, 2019 [29]	Turkey	Forest School		35	4–6				Drawings and interviews	Forest school can reshape the school
Quibell, 2017 [30]	UK	Wilderness Schooling	Matched-groups	244 (222) “218”	8–11	42		1	Attainment scores	OL improves children’s learning
Romar, 2019 [31]	Finland	Outdoor learning		21 (10) “11”	5–11				Acc; observation	OL is an effective complement to traditional classrooms
Schneller, 2017 [32]	Denmark	Teachout/EOtC	QE	361 (221) “140”	10.9	180	5	2	Acc	For boys, EOtC was associated with increased daily PA
Scott, 2016 [33]	UK			379	9–11				Written and science assessments.	OL can result in learning benefits across the curriculum
Trapasso, 2018 [34]	UK	Forest School	Mixed-methods intervention	59 (26) “33”	8.2	180	24		Acc; PAQ-C; write and draw	Nature-based learning within the curriculum increases PA
Yıldırım, 2017 [35]	Turkey		QE	35 (7) “28”	4.8–5.5	180	5	5		OL improves cognitive, linguistic, social-emotional, and motor skills

Acc, accelerometer; BOTMP, Bruininks–Oseretsky test of motor proficiency; DOSC, Danish Occupational Social Class; DAET-R, Draw an Environment Test Rubric; F, female; M, male; PA, physical activity; OL, outdoor learning; QE, quasi-experimental; SDQ, Strengths and Difficulties Questionnaire; UK, United Kingdom; USA, United States of America; VSMS, Vineland Social Maturity Scale.

**Table 2:** Standard Operating Procedure for a natural movement-based intervention.

Before		
Creation of the Team	Researchers Governments Schools Landscape Manager	
Training	Teachers Educators	Child has to feel safe Child has to be free Ownership of spaces Child has to manage the risks
During		
Performance of the project Nature used to learn Movement used to learn	Children	Length: 180 days Frequency: 2 times a week Duration: 2 h each session
After		
Evaluation	Teachers Educators Children	Physical activity level Learning outcomes

## Data Availability

Data used during the current study are available within the review.
